# Editorial: Recent advances in refractive surgery

**DOI:** 10.3389/fmed.2023.1270240

**Published:** 2023-08-15

**Authors:** Kazutaka Kamiya, Kazuno Negishi

**Affiliations:** ^1^School of Allied Health Sciences, Kitasato University, Kanagawa, Japan; ^2^Department of Ophthalmology, Keio University, Minato, Tokyo, Japan

**Keywords:** refractive surgery, SMILE, ICL, LASIK, cataract surgery

Recently, there has been swift advancement in the realm of refractive surgical methodologies, resulting in the emergence of refractive procedures that are both enhanced in safety and heightened in efficacy. New developments in surgical methodologies, including laser *in situ* keratomileusis (LASIK) and small incision lenticule extraction (SMILE), along with the implantation of phakic intraocular lenses (pIOL) and refractive lens exchange, aimed at correcting myopia, hyperopia, and astigmatism, have demonstrated significant promise in delivering optimal levels of safety and effectiveness concerning visual and refractive results. Moreover, these innovations strive to optimize visual acuity and consequent patient satisfaction. With the Research Topic on Frontiers in Medicine titled “*Recent advances in refractive surgery*”, we gathered new sophisticated surgical approaches on state-of-the art technologies poised to contribute to the ongoing progression within the field of refractive surgery.

The advent of SMILE over the past decade has introduced a viable substitute to LASIK for individuals seeking cornea laser refractive procedures. SMILE presents an innovative technique, relying exclusively on the femtosecond (FS) laser to shape an intrastromal lenticule that can be extracted through a minor incision. Presently, SMILE has solidified its status as a mainstream option among refractive interventions, catering to the management of mild to moderate myopia and myopic astigmatism. In a pioneering study, Han et al. undertook an initial bibliometric assessment of SMILE surgery spanning the last 12 years. Their findings revealed that research terminology associated with SMILE can be categorized into six distinct clusters: femtosecond (FS) laser technology, dry eye, biomechanics, visual quality, complications, and hyperopia, and that these words are helpful for better understandings of major concerns of surgeons and researchers in consideration of the further development of SMILE surgery.

Based on the fact of the lack of an eye tracking system as well as a control of ocular cyclotorsion in current SMILE surgery, there are still ongoing concerns about astigmatic correction, especially in high astigmatic eyes. Cui et al. demonstrated, in their meta-analysis selecting 6 randomized clinical trials for high astigmatism of 2.0 D or more, that SMILE, FS-LASIK and photorefractive keratectomy demonstrated similar effectiveness and safety profiles in correcting high myopic astigmatism. However, it should be noted that SMILE induced more residual astigmatism and diminished precision in forecasting astigmatic outcomes, alongside reduced surgically induced spherical aberrations when compared to alternative surgical methods. These findings indicate that the astigmatic nomogram requires some adjustments, especially in consideration of a slight tendency toward undercorrection for high astigmatism.

The posterior chamber pIOL (EVO Visian ICL) is widely acknowledged on a global scale as a long-term safe and effective approach for correcting moderate to high ametropia. Nonetheless, a low vault may be linked to cataract development, while a high vault can lead to angle closure and consequent elevation in intraocular pressure. Consequently, the ability to anticipate the ideal ICL vault assumes paramount significance in the realm of clinical practice, particularly when prioritizing the safety of ICL surgery. At present, the majority of surgeons rely on the manufacturer's sizing nomogram derived from measurements like white-to-white and anterior chamber depth, or utilize predictive formulas grounded in the data acquired from anterior segment optical coherence tomography (AS-OCT), in order to ascertain the most suitable ICL size. Wu et al. showed, using both AS-OCT and ultrasound biomicroscopy (UBM), that ICL size, ATA, and CLR, and ciliary sulcus angle (CSA) were essential for obtaining the optimal ICL vault, and that this derived formula outperformed the currently available NK and KS formulas. The combination of AS-OCT and UBM may hold a promise for further refinement of the optimal ICL size selection.

Contemporary cataract surgery has garnered recognition as a form of refractive surgery due to the incorporation of optical biometry and the latest-generation IOL power calculation formulas. However, it is still difficult to accurately determine IOL power in eyes undergoing corneal refractive surgery, possibly due to a keratometric measurement errors as well as effective lens position prediction errors. In their study, Wang et al. observed a strong correlation between alternations in standard keratometry (SK) and total keratometry (TK), as measured by swept source OCT (IOL Master 700), following SMILE and FS-LASIK procedures. These changes were found to be closely associated with shifts in spherical equivalent at the corneal plane, exhibiting a high correlation coefficient of 0.97. Notably, the authors introduced a novel approach by combining telecentric keratectomy with anterior segment optical coherence tomography (AS-OCT) to simultaneously measure the anterior and posterior corneal surfaces, leading to a more accurate prediction of intraocular lens (IOL) power after myopic corneal refractive surgery compared to SK. This enhanced precision could be attributed to the fact that the anterior/posterior corneal radius (A/P) ratio deviates further from the normal range, and also due to the refined alignment of TK with SK. The novel TK parameter could potentially offer a precise and objective assessment of corneal power that closely tracks the alterations in refractive change in corneal refractive surgery, thereby facilitating the attainment of a more exact refraction outcome.

In conclusion, this Research Topic provides us essential insights on refractive surgery for further refinement and offers the opportunity to contribute to the ongoing advancement of refractive surgical techniques. Modern refractive surgery has been become acknowledged as one of the most sophisticated surgical techniques in the field of ophthalmology.

## Author contributions

KK: Writing – original draft. KN: Writing – review and editing.

